# Machine Learning to Advance Human Genome-Wide Association Studies

**DOI:** 10.3390/genes15010034

**Published:** 2023-12-25

**Authors:** Rafaella E. Sigala, Vasiliki Lagou, Aleksey Shmeliov, Sara Atito, Samaneh Kouchaki, Muhammad Awais, Inga Prokopenko, Adam Mahdi, Ayse Demirkan

**Affiliations:** 1 Section of Statistical Multi-Omics, Department of Clinical and Experimental Medicine, Guildford GU2 7XH, Surrey, UK; rafaella.sigala1@gmail.com (R.E.S.); v.lagou@surrey.ac.uk (V.L.); a.shmeliov@surrey.ac.uk (A.S.); i.prokopenko@surrey.ac.uk (I.P.); 2 Surrey Institute for People-Centred Artificial Intelligence, University of Surrey, Guildford GU2 7XH, Surrey, UK; sara.atito@surrey.ac.uk (S.A.); samaneh.kouchaki@surrey.ac.uk (S.K.); muhammad.awais@surrey.ac.uk (M.A.); 3 Centre for Vision, Speech and Signal Processing, University of Surrey, Guildford GU2 7XH, Surrey, UK; 4 Oxford Internet Institute, University of Oxford, Oxford OX1 3JS, Oxfordshire, UK; adam.mahdi@oii.ox.ac.uk

**Keywords:** genome-wide association, human genetics, machine learning

## Abstract

Machine learning, including deep learning, reinforcement learning, and generative artificial intelligence are revolutionising every area of our lives when data are made available. With the help of these methods, we can decipher information from larger datasets while addressing the complex nature of biological systems in a more efficient way. Although machine learning methods have been introduced to human genetic epidemiological research as early as 2004, those were never used to their full capacity. In this review, we outline some of the main applications of machine learning to assigning human genetic loci to health outcomes. We summarise widely used methods and discuss their advantages and challenges. We also identify several tools, such as Combi, GenNet, and GMSTool, specifically designed to integrate these methods for hypothesis-free analysis of genetic variation data. We elaborate on the additional value and limitations of these tools from a geneticist’s perspective. Finally, we discuss the fast-moving field of foundation models and large multi-modal omics biobank initiatives.

## 1. Introduction

Genome-wide association study (GWAS) is a hypothesis-free statistical approach for assessing associations between genetic variants and phenotypes in a sample population [1]. To date, more than 60,000 genetic associations have been reported in more than 6000 GWASs [2] with their summary statistics being publicly available in repositories, such as the GWAS Catalog [3], GWAS Atlas [2], and Roslin gene atlas [4], among others. Although the most popular area of GWAS application has been human genetics, this approach has also been successfully applied in genetic research for fungi [5], bacteria [6], plants [7], and animals, including wild and companion animals [8], as well as livestock populations [9], proving its widespread use across agricultural, veterinary, and medical sciences. GWAS not only have been instrumental in discovering genetic variants as potential causal factors for human diseases but also encouraged the development of genotyping platforms and new statistical methods, as well as investment into huge DNA biobanks with petabytes of phenotype and genotype data from various ethnic groups [10].

The main ambition and outcomes anticipated when designing GWASs has been two-fold. First is to understand the biological pathogenesis of human diseases or variation in quantitative traits, such as height or endophenotypes. Such knowledge can be used for the development of disease therapeutic options by blocking the downstream path of a culprit molecule, or by replacing the missing molecules. Second, identify the individuals at risk of a certain disease, often by calculating polygenic risk scores (PRS). In the case of quantitative traits, the quantitative prediction is translated into a liability threshold, (e.g., BMI > 30) to predict obesity.

### 1.1. The Road from GWAS Findings to Drug Discovery

GWAS for several diseases have led to the identification of a large number of associated variants in functionally plausible genes as in the case of *FTO* for obesity [11], *SLC30A8* for type 2 diabetes [12], and *APOE* for Alzheimer’s disease [13]. In a more recent GWAS meta-analysis, missense variants in the *GLP1R* locus with significant effects on random glucose were functionally followed up [14]. It was shown that some of these variants responded differently to GLP-1R agonist drugs, commonly used in managing diabetes, indicating the importance of tailored treatments based on genetic variability.

Several examples of therapeutically actionable GWAS variants, which map to genes modulated by currently used drugs for diseases, have been described [15]. Causal evidence from human genetic studies prioritizing genes encoding approved drug targets or their interacting proteins correlates with higher success rates in clinical trials [16]. The translation of GWAS signals into therapeutic targets requires the integration of multiple omics layers, as well as clinical knowledge of the pathophysiology of the disease. Open-source informatic solutions can assist in the identification and prioritisation of targets. For example, Open Targets Platform aggregates evidence scores from 22 different data sources capturing information from genetic associations, somatic mutations, known drugs, affected pathways, literature mining, differential expression, and animal models [17]. Among these data sources, Open Targets Genetics aims to overcome the challenges of identifying the most likely causal variant and the actual causal genes at each GWAS locus for common, complex traits/diseases by integrating genetic and functional genomics features [18]. The application of complex statistical models on larger studies with broader phenotyping and better knowledge of disease pathophysiology offer opportunities not only for de novo drug development but also drug repurposing. However, most GWAS signals do not present large enough effect sizes to be translated into drug targets, apart from some notable exceptions, such as *APOE* for Alzheimer’s disease [19].

### 1.2. GWAS Applications beyond Gene Discovery: Cumulative Genetic Profiles and Causal Relationships

GWAS findings demonstrated that most common non-communicable diseases show high polygenicity with each individual associated variant, accounting for a small proportion of phenotypic variance. It set the floor for more efficient identification of individuals at high/low disease risk by calculating PRS and summing the weighted effect size of each associated variant [20]. PRS have been first introduced for highly polygenic mental disorders for which initial GWAS underachieved [21,22]. Later, they were constructed for various diseases, such as coronary artery disease, hypercholesterolemia, and T2D [23]. Although it improved disease prognosis compared to conventional risk factors, their value in clinical practice is still questionable highlighting the existing challenges [24]. For example, PRS studies can face ancestry biases with limited transferability across populations due to differences in risk allele frequencies, heritability, linkage disequilibrium, and clinical heterogeneity [25]. The majority of existing PRS have been constructed based on variants identified in European populations. These Eurocentric PRS might be less predictive for other ethnic groups with substantially lower allele frequencies for these variants. Furthermore, certain modifiable factors, such as diet, alcohol consumption, smoking, and physical activity, correlate with genetic ancestry influencing phenotypic variance and PRS accuracy [26]. Even within populations of the same ethnicity genetic differences are present leading to bias when PRS are trained and tested on different subpopulations [27]. Overall, more advanced methods are necessary to improve risk prediction models, making PRS implementation into clinical practice a reality.

Additionally, GWAS results fuelled the development of novel approaches enabling discoveries of the complex relationships between human traits, exosomal, and intrinsic factors. Among the most popular approaches is Mendelian Randomisation, a method powered by a plethora of GWAS data to estimate the causal effect of exposure on an outcome dissecting the causal relationships between phenotypes [28]. Mendelian Randomisation relies on effect estimates and standard errors obtained from individual SNPs in either single GWAS or through meta-analysis of GWAS. Finally, the genetic correlation between two different phenotypes is not necessarily measured on the same individuals and can be calculated by using GWAS outputs [29].

In GWAS, a separate statistical test is performed for each SNP and the identified individual variants only account for a small proportion of the heritability of complex diseases and traits. This is partially due to a lack of robust methodology for studying SNP-SNP interactions. Typically, GWAS analysis requires a large sample size for statistical power, which is achieved by meta-analysis of hundreds of GWAS [30] conducted on distinct populations [31]. Unfortunately, GWAS findings often lack direct biological interpretation and post-GWAS methods are necessary for drug development.

## 2. Machine Learning Solutions for GWAS

Machine learning, a subfield of artificial intelligence, deals with the development of algorithms capable of learning from the data. Recently, the application and development of machine learning methods for genomics have undergone rapid growth. It proved valuable for analysing complex, high-dimensional genomics data and extracting previously unknown information. Examples of machine learning applications in the wider omics field range from the identification of DNA sequences (splice sites [32], promoters [33], enhancers [34]), nucleosome positioning [35], taxonomic annotation [36], microbial enterotyping [37], sequence errors learning [38], microbial host body site and subject classification [39], viral escape prediction [40], protein 3D structure estimation [41], evolutionary population genetics inference [42], and genomic selection [43].

### 2.1. Machine Learning Methods Frequently Adapted for GWAS

PubMed and Google Scholar were searched for journal articles that included the keywords “machine learning” and “genome-wide association study”. We focused on papers written in English and published from 1 January 2004 to 6 November 2023. An initial set of 147 articles was selected and then reviewed based on their title, keywords and abstracts for inclusion. Papers that did not match the inclusion criteria were eliminated, resulting in 109 articles. We then assessed the full text of those papers, which were further categorised based on their context and relevance including research articles that applied machine learning algorithms to GWAS, PRS, and review papers. We also included benchmarking research which used real data excluding the ones that used only synthetic data. From this set of articles, duplicate papers were also deleted. This resulted in 79 relevant papers, of which 60 were research articles and 19 review articles. The methodology in each research article was analysed to identify the specific machine learning tools and their unique features. The most common methods included Support Vector Machines (SVMs), random forests, and neural networks. We provide a short background for these methods below.

Random forest [44] is an ensemble learning method commonly used in GWAS. In a random forest, several weak classifiers (e.g., trees) are constructed, each using a random subset of the training data and a random subset of the features. This randomness in data and feature selection is a key element of the method, which mitigates the risk of overfitting and helps ensure the model’s generalisation to new, unseen data. Each tree in the forest independently makes predictions based on its specific subset of the data. When a new data point is presented to the model, it passes through each decision tree and their individual predictions are aggregated. In classification tasks, the final prediction is often determined by a majority vote among the trees, while in regression tasks, it is the average of the predictions. Random forests are particularly strong at handling high-dimensional genomic data commonly encountered in GWAS, providing insights into the importance of individual genetic features and interactions among them [45]. Random forests can also be used to perform feature importance rankings, helping researchers to identify key genetic variables contributing to complex traits, as discussed below.

SVMs [46] are a class of machine learning algorithms designed to classify data by identifying the optimal hyperplane that best separates different classes in a high-dimensional feature space. In the context of GWAS, SVMs map genetic data that is often represented as high-dimensional feature vectors in multi-dimensional space. The goal is to identify the hyperplane (decision boundary) that maximises the margin between different genetic variations associated with a particular trait or disease. SVMs work by selecting support vectors, which are the data points closest to the decision boundary. These vectors play a key role in determining the orientation and position of the hyperplane. The choice of the optimal hyperplane is critical because it minimises the risk of overfitting and aims to generalise well to unseen data. SVMs can also handle non-linear relationships through kernel functions, transforming the input data into a higher-dimensional space, where a linear separator becomes feasible.

Neural networks [47] rapidly gained significance in GWAS, mainly due to their ability to uncover complex genetic patterns within high-dimensional genomic datasets. The basic building block of a neural network is the artificial neuron (also referred to as a node). Each neuron transforms input data through a weighted sum, which is followed by the application of an activation function. By connecting neurons in layers, neural networks can model increasingly abstract and complex relationships. In the context of GWAS, these networks are often designed as deep neural networks [48,49] with multiple hidden layers, to extract hierarchical features from genetic data. Neural networks are especially suited at capturing non-linear relationships among genetic variants [49]. During the training process, they adjust their internal parameters to minimise prediction errors. This training process involves feeding the network with genetic data and adjusting its parameters until it can make more accurate predictions. Once the model has been trained, neural networks can be used for a variety of tasks, including classification, regression, and feature selection.

### 2.2. Machine Learning Application Areas in GWAS

In this section, we present the methods, benchmarking efforts, and specifically designed tools which integrate machine learning approaches working with high-dimensional genetic data, the results of which are promising in identifying novel disease-associated susceptibility loci. These studies suggest that machine learning could be used instead of traditional statistical GWAS methods, potentially aiding in the better understanding of complex multifactorial genetic diseases and the prediction of individuals at risk. Benchmarking efforts of using machine learning in the field of GWAS are mainly focused on four methods: gradient boosting, random forest, SVM, and neural networks. Here, we simplify the classification of applications by prioritising top GWAS SNPs and genes, detecting epistasis among selected loci, search space reduction, predicting traits, identifying variant/loci, and supporting PRS.

***Prioritization of top GWAS SNPs and genes.*** In GWAS, the multitude of input features (SNPs) often exhibit correlation due to linkage disequilibrium. This correlation leads to many SNPs having closely related *p*-values of statistical significance. Initially, the common approach was to prioritize the top trait-associated SNP and link it to the nearest gene. However, solely relying on physical proximity can be misleading as SNPs can affect gene expression across extensive genomic distances. This necessitates a more nuanced understanding and exploration of how genetic variations impact gene expression and function beyond just physical proximity [11]. Expression quantitative trait loci studies suggest that two-thirds of the causal genes at GWAS loci are not the closest [50,51]. Thus, identifying the most likely causal SNP is a challenge in GWAS. Paired with functional validation, machine learning shows important promise for clinical translation, providing a strong evidence-based approach to direct post-GWAS research. Machine learning applications developed for post-GWAS prioritisation (up until 2020) were summarised by Nicholls et al. [52] who pointed out that 7 out of 19 post-GWAS prioritisation methods were ensemble methods, namely gradient boosting and random forest.

An example of how neural networks can be used to prioritise disease-associated genetic variants can be found in Liu et al. (2018) [53]. They developed DEOPEN, a model which integrates a deep convolutional neural network and a three-layer feed-forward neural network. This model can predict chromatin accessibility and consider interactions between sequence patterns. The authors also demonstrated how their framework can be used to evaluate genetic variants of interest, including functional variants. The authors applied their framework to a GWAS breast cancer GWAS data which identified 29 SNPs associated with this condition from 1057 SNPs that co-occurred with them, through their involvement with a cancer-related transcription factor, FOXA1.

The remaining methods focus on prioritisation of genes, rather than SNPs. Open Targets recently introduced new techniques for prioritising GWAS results [54]. Their “locus-to-gene” model derives features to prioritise likely causal genes at each GWAS locus, incorporating genetic and functional genomics features such as distance, molecular QTL colocalization, chromatin interaction, and variant pathogenicity. The locus-to-gene method uses a machine learning model to determine the weights of each evidence source, referencing on a gold standard of previously identified causal genes and relying on fine-mapping and colocalisation data.

A random forest-based classifier, GCDPipe [55], uses gene-level results derived from GWAS analysis. It expands the list of potential disease gene candidates through the estimation of probability to influence disease risks. GCDPipe identifies gene expression profiles across cell types and tissues with the highest importance for putative disease gene identification. Additionally, it prioritises drugs based on affinity to the putative disease genes using drug-gene interaction databases.

One remarkable benchmarking effort for prioritisation of causal genes was done by Vitsios and Petrovski (2020) [56] and compared seven different machine learning methods to prioritise genes for amyotrophic lateral sclerosis, chronic kidney disease, and epilepsy. They implemented a diverse pool of gene-annotation sources: generic resources (disease and/or tissue agnostic) and resources filtered by tissue and disease-specific features. They also developed “mantis-ml” as an automated machine learning framework to enable learning from sets of gene-associated features. Random forest was reported as the top-performing classifier.

***Epistasis detection among selected loci.*** Random forest was initially suggested as an alternative to model genetic interactions in 2004 [45]. The rationale behind employing random forest is that in situations involving genuine interactions, SNPs exhibit modest individual effects but considerable interaction effects within a population. However, such effects are less likely to be detected at the genome-wide multiple testing thresholds used in GWAS screenings. Moreover, model-based screens that assess the interaction of each SNP with every other SNP in the dataset, aiming to pre-specify interacting SNPs, are impractical for datasets exceeding a thousand SNPs. Given that a typical GWAS dataset usually comprises more than 50,000 SNPs, such an approach becomes unfeasible.

Random forest analysis of interacting genetic models, up to 32 independent SNPs showed that random forest performed better than Fisher’s exact test as a screening tool when genetic heterogeneity as well as random noise is accounted for. In this study, the authors recommended that thousands of trees must be used in order to get stable estimates of the variable importance [45]. An advantage of random forest is that the investigator does not need to propose a model, making it well-suited for hypothesis-free screens such as GWAS or candidate gene studies. It also captures interactions and reflects them in variable importance scores. Drawbacks of the method include lack or concordance between variable importance and predictive index value [57] and the high chance of detecting false, spurious associations when the study design is sub-optimal [58].

In 2015 Nguyen et al. [59] developed ts-RF which is a two-stage method for selecting informative SNPs, i.e., a small portion of the SNPs that have main effects on the disease. In this method, first, a *p*-value assessment is performed to find a cut-off point that separates the genome-wide SNP data into relevant and irrelevant SNPs. The informative SNPs group is further divided into two sub-groups: highly informative and weak informative. Then, these two groups are considered when sampling for building trees. So, the feature subspace is encouraged to contain highly informative SNPs when used to split a node at a tree, resulting in better performance in RF. They applied ts-RF to real genome-wide datasets of Alzheimer’s and Parkinson’s disease and compared its performance of linear kernel SVM from LibSVM [60]. ts-RF performed better at prediction and was able to point to 25 SNPs associated with Parkinson’s disease that are located within gene regions studied by previous GWAS.

A recent report described by Leem et al. [61] suggested a three-step approach allowing authors to define up to 5-locus interactions in real WTCCC datasets and synthetic datasets without marginal effects. In the same study, they also proposed and tested the use of *“mutual information value”* as the measure of association between genotypes and susceptibilities of diseases, for extracting the SNPs which engage in interaction. Also, there have been multiple attempts to find interacting genetic loci by other machine learning methods, such as decision trees (DF-SNPs) [62], Deep Mixed Model [63], and grammatical evolution optimised neural networks (GENN) [64].

***Search space reduction*** One important area of machine learning for GWAS has been to reduce the search space for following analyses or by prioritising loci to be included in GWAS subsequently by increasing the *p*-value threshold and power in detecting significant loci. To this end, stand-alone but also combinatory tools have been developed for search space reduction.

Silva et al. [65] showed that dimensionality reduction techniques based on random forest could effectively reduce dataset dimensions before conducting a cluster analysis of augmented GWAS data using a two-step machine learning approach. In the first step of dimension reduction, SNPs were ranked based on their relevance, and those with higher relevance underwent the second stage of analysis, which involved clustering. They tested the method on seroclearance GWAS in chronic hepatitis B while including the most significant SNPs in the clustering. The results included over 100 SNP sets which were associated with the phenotype of interest. SNPs were further detected and linked to HBsAg seroclearance with statistical significance based on Hamming distance-based association tests [66] in which a *p*-value for each predetermined causal SNP set was calculated. Knowing that statistically significant variants tend to cluster, the authors also investigated the functional relevance of SNPs found in the same SNP-set, as well as in individual SNPs followed by random forest and identified possible susceptible loci that could be otherwise ignored when only performing GWAS. The resulting SNP-sets from the cluster analyses were subsequently tested for trait association and identified three susceptibility loci possibly associated with HBsAg seroclearance one of which was reported in the literature to be significantly associated with HBsAg seroclearance in patients who had received antiviral treatment.

Random forest was further combined with SVMs and k-nearest neighbour (kNN) clustering methods [67] by Gaudillo et al. and used for asthma genetic risk prediction. In their study, they applied random forest to identify the SNPs highly implicated in asthma. Following that, they trained kNN and SVM algorithms to classify the identified SNPs for their association to asthma.

New frameworks using SVMs continue to be developed, while their performance is also shown to be heavily influenced by the heritability of the disease [68].

Recent research in Alzheimer’s disease [69] used a hybrid feature selection approach based on association test, principal component analysis and the Boruta algorithm, to identify the most promising predictors. The selected features are then forwarded to wide and deep neural network models to classify the Alzheimer’s disease cases and healthy controls. In the first step, they conducted an association test to select the most signification SNPs influencing the disease, followed by a hybrid feature selection approach to reduce the number of features substantially. They subsequently used a selection process for neighbouring SNPs to generate a final set of SNPs. This set was then used to train wide and deep learning classification models for both cognitively normal individuals and those with Alzheimer’s disease.

Another method is DeepGWAS which uses a 14-layer deep neural network to enhance power in GWAS signals without increasing the sample size, by assigning unequal a priori probability for each SNP involvement in disease leveraging linkage disequilibrium information and brain-related functional annotations. DeepGWAS was developed particularly for psychiatric diseases, starting with schizophrenia and outperformed XGBoost and logistic regression methods [70]. COMBI [71] and DeepCOMBI [72] also have built-in ML-based variant prioritisation functions which are discussed in more detail below. The range of applications using combinatory approaches continues to expand (Table 1).

### 2.3. Tools for SNP Discovery from Whole-Genome SNP Data

There is a growing number of efforts that use SVMs and neural networks to narrow down the search space for GWAS. Additionally, there are tools designed to perform GWAS with no prior hypothesis or feature selection. Below, we discuss algorithms and publicly available tools which have undergone internal benchmarking but warrant further testing in broader genetic epidemiological research (Table 2).

***COMBI* [71]**. A method by Mieth et al. (2021), COMBI [71], employs a linear SVM which is trained and used as an indicator of importance and SNPs from each chromosome separately. This first filtering step selects SNPs which contribute to phenotype classification with either high individual effects or effects in combination with the rest of SNPs while removing results due to the correlation structure. At the application level, a phenotype vector and a genotype matrix which can be directly converted from a Plink [80] genotype object are generated. From these two objects, the SVM weight vector is generated and used as an importance measure. In the second step, SNPs with the higher scores selected undergo a chi2-based hypothesis test performed together with Westfall-Young [81] type threshold calibration for each SNP, based on the permutation distribution of the re-sampled *p*-values. In this way, using a pre-selected list of SNPs and a relaxed *p*-value threshold the proportion of true positives in the data is ultimately increased. In the simulated dataset COMBI overperformed other SVM-based algorithms, including those previously mentioned by Roshan et al. [82]. Following that, they used data from the 2007 WTCCC phase 1, consisting of 14,000 cases of seven common diseases and 3000 shared controls. When compared to the standard *p*-value thresholding approach, COMBI detected twelve additional SNP, ten of which have already been replicated in later GWAS or meta-analyses of bipolar disorder, coronary artery disease, Crohn’s disease, and for type 2 diabetes.

***DeepCOMBI* [72]**. The authors of COMBI subsequently developed a “deep” extension of COMBI, called DeepCOMBI [72]. This extension was designed to identify SNPs associated with a trait of interest, leveraging genotypic and phenotypic data from GWAS. The methodology includes the construction of deep neural networks for phenotype prediction of any genotype and SNPs selection according to a threshold, followed by layer-wise relevance propagation application on the SNPs and the selection of the most relevant variants. Lastly, a hypothesis test is performed for each variant. In addition, layer-wise relevance propagation yields the relevant scores for each variant and the permutation test can guarantee the selection of novel SNPs based on their *p*-values. In their report, DeepCOMBI showed a better performance compared to other methods and identified a higher number of significant SNPs with the lowest error rate.

***GenNet* [73]**. Applying fully connected networks to millions of SNPs requires an ample amount of computational time and memory. To overcome these limitations, developers of GenNet provided a novel framework for predicting phenotype from genotype [73]. GenNet uses neural network, as well as prior biological knowledge, to create groups of nodes that are connected among the layers, reducing the sum of learnable parameters that a fully connected neural network would need. Biological knowledge may include information on gene annotation, local and global pathways, exon annotation, chromosome annotation, as well as cell and tissue type expression. In this model, neurons represent biological entities, and the weights signify the effects between neurons, resulting in a biologically interpretable network. This method allows human biological input, via a straightforward framework with help of two other pieces of software, HASE [83] and ANNOVAR [84], embedded in for generating necessary files. The major drawback of the method is that any researcher can perform differently layer annotation, making it difficult for standardisation.

***GMStool* [75]**. The tool was developed and tested on soybean but can be easily applied to human GWAS with no modification. Overall workflow consists of three phases: preparation, marker selection, and final modelling. The preparation phase includes preparation of data which are genotype matrix, phenotype file, and a GWAS summary statistic file as the training set. The marker selection phase applies the forward selection method of regression analysis and sequentially selects SNP markers that increase the correlation rate between observed and predicted phenotypes on the validation set. The ridge regression best linear unbiased prediction and bootstrap trees methods are provided as learning models. The final modelling phase performs prediction modelling using ridge regression, random forest, deep neural network, and convolution neural network models, using either only one of them, or all. Unfortunately, the current construction of the GMStool requires the use individual level data in addition to GWAS summary statistics, limiting the application areas of the method.

***Deep Mixed Model* [63]**. GWAS on moderately or cryptically related individuals have customised methods to correct for relatedness, usually either by genetic components or mixed models. To account for relatedness in genome-wide deep learning applications Wang et al. [63] proposed a Deep Mixed Model which consists of two components. The first component (the corrector) acts as a confounding factor correction by using a convolutional neural network and further calculates the residual phenotype after removing confounding effects. The second component (selector) uses Long-short Term Memory for genetic variants selection, to identify the SNPs that are associated with the residual phenotype in univariate, polygenetic, or epistastic manners. Six out of twenty SNPs selected by the Deep Mixed Model were annotated to genes associated with Alzheimer’s disease.

***GWANN* [74]**. Ashkenazy et al. (2022) [74] tried to exploit the ability of convolutional neural networks in image recognition by developing and training a method for the classification of variants associated with a trait of interest, using genomic data converted to image patterns. The model named GWANN, was trained using true positives and true negative data corresponding to trait association and finally makes prediction in a tested population. GWANN performance deteriorated when the simulated population did not accurately represent the tested data. For example, a minor allele frequency of less than 5% affected the pattern of SNP images, influencing the model’s sensitivity. Therefore, parameters such as minor allele frequency, population structure, population size, and sampling rate in the training populations need to be adjusted.

***DeepWAS*** [85]. A multivariate functional unit-wide association study (DeepWAS) was developed with the aim to only include SNPs that have been prioritised based on their risk potential. Genome-wide SNPs are first analysed for their functional roles and their association with specific cell lines and transcription factors using the deep learning model DeepSEA [86]. DeepWAS was able to identify and validate novel disease-associated loci in multiple sclerosis, major depressive disorder and height that could not be identified in smaller cohort GWAS studies. It was also able to identify associations of SNPs within a functional unit relevant to a trait that is typically missed in traditional GWAS. This methodology is ideal for any GWAS dataset if disease-associated genetic conditions (cell-type effects, chromatin features) and its functional data are available. DeepWAS reduces the multiple testing burden of classical GWAS and makes regulatory information on a single SNP level readily available without requiring a second analysis step.

***iMEGES*** [76]. Integrated Mental-disorder GEnome Score (iMEGES), this method was developed as a deep learning tool for analysing whole genome/exome sequencing data, primarily for mental disorders [76]. In the first step, iMEGES prioritises variants based on non-coding and coding variants using tools EIGEN, CADD, DANN, GWAVA, FATHMM, known brain eQTLs from CommonMind, and enhancer/promoters from PsychENCODE and Roadmap Epigenomics projects. In the second step, genes are prioritised based on annotations for each variant from the first step of iMEGES. Table 2 shows an overview of practical properties of these tools which are only internally benchmarked, requiring parallel assays for evaluating their analytical power over each other

### 2.4. Applications Supporting PRS

While standard PRS is built upon linear models, below we summarise three methods which used nonlinear approaches to support disease prediction by GWAS based PRS. In the breast cancer study by Badre et al. [78], the authors used a deep neural network for breast cancer prediction and compared it to established statistical algorithms, via a combinatory design; first selecting SNPs by Plink and then building PRS either by deep neural network which they called neural network risk score or linear methods. Deep neural networks outperformed the best linear unbiased prediction methods [87].

Zhou et al. [77] developed deep neural network models for modelling Alzheimer’s disease polygenic risk and compared them with the widely used weighted PRS and LASSO models. In their study, they first selected the disease-associated SNPs from a GWAS summary statistics and then predicted three different scenarios of training/validation splits. They considered the biological properties of variants, including gene and functional chromatin annotations, to build seven-layer neural networks. Not the neural network risk score performs slightly better than weighted PRS and LASSO, but it is also significantly associated with levels of the blood-based biomarkers of disease pathology.

Tree-based statistical learning methods were also tested for better PRS construction [79], showing that random forest and logic bagging outperform other tree-based (logic regression, elastic net, and RF-VIM) methods for predicting rheumatoid arthritis.

In machine learning analyses followed by statistical evaluations of single SNPs, the initial step involves selecting a set of SNPs based on their relevance scores. Tools like COMBI, deepCOMBI, GenNET, and iMEGES possess built-in functions to derive these relevance scores. Consequently, these methods are more interpretable and explainable at the single SNP level. In contrast, other tools primarily focus on prediction modelling without providing explicit SNP relevance scores, differing in their architecture and intended use.

One particular application area emerged as GWAS of image-derived phenotypes, e.g., from optic nerve photographs and magnetic resonance imaging [88] as distinct measures of brain structure and function. Aggregating the complex geometric and topological structures present in images into biomarkers that are useful in a GWAS setting is a challenge. Methods such as *transfer*GWAS [89] and iGWAS [90] to improve retina images, optic nerve head [91], as well as employing convolutional neural networks to improve brain imaging endophenotypes [92].

## 3. Limitations and Criticism of Machine Learning

While machine learning offers plethora of new tools when combined with countless combinations of multi-modal omics data, there are multiple concerns for its use in GWAS.

***Exploitability***. As previously mentioned, the primary use of GWAS has been to understand the biological factors underlying human traits and diseases, at the single nucleotide resolution. To this end, machine learning methods have only focused on prediction, which cannot be used to identify molecular drug targets by default. However, the same methods can be very powerful in predicting and classifying diseases. Recently, there has been considerable research dedicated to developing interpretability frameworks toward hypothesis-free genome scans [73]. Applications such as GenNet and iMEGES are promising tools as their methods largely benefit from functional annotations across the human genome.

***Comparability.*** So-called interpretable machine learning applications provide feature importance scores reflecting the importance or relevance of variables in the prediction model [73]. However, they can neither be translated into effect estimates nor *p*-values which constitute the summary statistics tables in large repositories. Thus, there is limited comparability between data accumulated in conventional GWAS repositories and those generated by machine learning.

***Standardisation and data accumulation***. GWAS methodology has been developed via rigorous consortia work for almost two decades. Standards related to study design, sample size, replication, population stratification, and meta-analyses have been integrated into practical workflows. Currently, there is a lack of standardisation for best practices in applying machine learning to human genetics. Since the field is still in its early stages, it requires guidelines to define the best approaches.

***Data imbalance***. When employing machine learning in GWAS, an often overlooked issue is data imbalance. These methods typically require an equal number of cases and controls [73], yet most biobanks, designed in population-based settings, have significantly fewer cases than controls. While techniques like adjusting loss function and under-sampling can address imbalance to some extent, their application in large biobanks is limited. This may influence future study design choices in biobank collections. However, similar to GWAS, machine learning also faces limitations in study power, heavily reliant on sample size [93] and disease heritability [68].

***Ethical issues.*** Gaps between the design and operation of algorithms and our understanding of their ethical implications can have severe consequences affecting individuals as well as groups and whole societies [94]. Issues currently present in large genetic association studies, e.g., ethnicity, gender and socio-demographic bias will extend themselves to the field AI as well [95]. One remarkable example in health care is the so called “skin cancer algorithm” which was be developed on datasets that under-represent darker skin types, which may exacerbate the health disparities of some geographic regions [96]. When applied in nation-wide health care setting, such algorithms not only prone to major ethical problems but also, they will perform less optimum in certain sub-populations, influencing the liability and security of AI as well. This is also related to the unfairness in data access, sharing and transparency of AI algorithms. Accessibility to algorithms should be provided as wells as information on how they work. Transparency on how the algorithm functions is necessary create a form of trust between those who design the tools and the ones testing and using them, which is important for future collaborations for AI tool development [97].

## 4. Future Prospects

Here we emphasize the two important drivers of the field, growing numbers of biobanks and fast developing new AI methods.

### 4.1. Multimodal Omics Databases

One of the most important applications of machine learning in the medical field is the development of multimodal AI models necessary for the integration of omics data across different modalities from biobanks and initiatives [94]. These studies are designed to include hundreds of thousands of individuals with in-depth genetic and health information that are regularly enriched with new omics layers and follow-up measurements. The data generated are high-dimensional and multi-layered as they incorporate a massive collection of “omics” (genomic, transcriptomic, proteomic, metabolic, or microbiome) along with electronic health records and study-specific other measurements. The best-known longitudinal population-based biobanks include the UK Biobank [98], the China Kadoorie Biobank [99], the Estonian Biobank [100], and the Lifelines Biobank [101]. The use of this data through the implementation of AI methods has allowed high-throughput analysis and has led to new discoveries in the medical field [69] and shown to improve prediction in comparison to an unimodal approach [94].

### 4.2. Opportunities of Large Language Models and Foundation Models

Genomic sequences are vast repositories of complex biological data containing distant semantic relationships which may not be fully captured by traditional AI methods although ideal for foundation models. In traditional AI, most of the computing resources were spent on training models for specific tasks. To train such models, we need large amounts of labelled data (e.g., outcomes) which is often expensive, especially in the healthcare field. On the other hand, foundation models are large deep neural networks pre-trained on diverse data from a range of problems using *self-supervised learning* [102,103], which does not require expensive human labels. Once these foundation models are pre-trained, they can be finetuned for downstream tasks which are specific to a particular problem using relatively little labelled data or in some cases no labelled data. Therefore, foundation models have been transforming the AI landscape in natural language processing, computer vision, and multimodal analysis including the field of omics. Foundation models started to emerge in natural language processing around 2018 and in 2023, multimodal foundation models appeared in healthcare and radiology [104].

The self-supervised learning principles which are behind these foundation models are usually based on simple principles. Typically, words are converted to a vector representation using simple neural network embeddings. Then, the job of the deep neural network in self-supervised learning is to recover words masked randomly from the context. For example, BERT [98] masks 15% of the words randomly and recovers these words at the output. In addition, BERT predicts whether two sentences are next to each other or not. On the other hand, GPT like model simply predicts the next word in the sentence. If the deep neural network is unable to predict the right words, their weights are updated using back propagation algorithms [105]. When applied in genomics, DNA or RNA strings can be considered as text documents with characters in DNA or words in proteins enabling foundation models to capture complex local and distant semantic relations.

The complexities of genetic information pose unique challenges, such as high dimensionality and the need for significant computational power, which have so far hindered the widespread adoption of foundation models in this area with relatively few publications applying basic concepts of foundation models to genomic data [104,106,107,108]. For example, Santiesteban et al. [109] showed that foundation models combining transcriptomics and histopathology data through self-supervised learning significantly improve survival prediction. As the volume of omics data continues to grow in biobanks and computational capabilities advance, the full spectrum of foundation models’ capabilities is likely to bring a new era of scientific discovery and innovation in biomedicine.

## 5. Conclusions

Broad range of applications under the machine learning umbrella offer solution for some of the problems in GWAS; however, application of these methods carelessly may also mitigate their benefits. We believe the benefits of this new interdisciplinary area will increase by building a common language and aims and through collaborative efforts, towards ethical, secure, and trustworthy AI applications.

## Figures and Tables

**Table 1:** An overview of machine learning tools classified by application categories and machine learning approaches.

Application Categories	Applications and Tools	Machine Learning Approach
Prioritization of top GWAS SNPs and genes	DEOPEN [53] GCDPipe [55] Mantis-ml [56] Open Targets [54] Methods developed prior to2021 [52]	Clustering SVM Random Forrest Neural Network
Epistasis detection among pre-selected SNPs	DF-SNPs [62] random forest [45] DEOPEN [53] K-means [61] ts-RF [59]	Clustering Random Forrest Neural Network
Search space reduction	clustering, random forest [65] random forest, SVM, kNN [67] Wide and Deep Learning [69] DeepGWAS [70] COMBI [71] DeepCOMBI [72]	SVM Random Forrest Neural Network
Hypothesis-free GWAS	COMBI [71] DeepCOMBI [72] Deep Mixed Model [63] GenNet [73] GWANN [74] GMStool [75] MACLEAPS [68] iMEGES [76]	SVM Neural Network
Polygenic Risk Score	NNP [77] DNN [78] RF-GRS [79]	Random Forrest Neural Network

**Table 2:** Currently available tools that are designed for outcome prediction or gene/SNP discovery from genome-wide variation data.

Name	Method	Genotype Matrix Generation	Explainability/Method for SNP Relevance Scores	Language
COMBI	Two-step method: (1) SVM training and selection of SNPs relevant for phenotype classification (2) Statistical testing	Not built-in. It requires a phenotype vector and a genotype matrix.	Yes/SVM for SNP relevance scores	Matlab/Octave, R and Java
DeepCOMBI	Three-step method: (1) Training of a DNN for classification of subjects into their respective phenotypes (2) Calculation of SNP relevance scores (LRP) and SNP selection (3) Statistical testing	Not built-in. It requires a phenotype vector and a genotype matrix.	Yes/relevance scores	Python
Deep Mixed Model	Two-component DL method: (1) One-dimensional CNN (confounding factor correction) (2) A LSTM model for selecting SNPs that contribute to residual phenotype in an epistatic manner	Not built-in. It requires genotype and phenotype matrices.	Not available	Python
DeepWAS	Integration method: (1) DL-based functional annotation of single GWAS SNPs for their regulatory effects on cell type-specific chromatin features (pre-trained DeepSEA network) (2) Association of regulatory SNPs with a disease/train into a multivariate setting (regularized regression models)	Not built-in. DeepSea requires vcf format.	Not available	R
GenNet	Use of NN with connections defined by prior biological knowledge to create groups of nodes across different layers to reduce the number of learnable parameters	Built-in	Built in as SNP, gene and pathway relevance scores based on relative weights	Python
GMStool	Three-step method: (1) Preparation of input files (2) Marker selection (RRB and/or BTS) (3) Prediction modelling (RRB, RF, DNN and/or CNN)	Not built-in. It requires genotype, phenotype, GWAS result and test list files.	Not available	R
GWANN	(1) SNP data is converted into a learnable image (matrix) (2) The constructed images, each representing a SNP, are classified as either associated or not-associated with the trait using a CNN.	Not built-in. It requires a VCF file with genotype data and a csv file with phenotype data.	Not available	Python
iMEGES	The Annovar input/bed format file	Not built-in. It requires various predictors for genotype data from ANNOVAR, BED or VCF files.	Built in.	Python

List of specifically designed tools for gene discovery or outcome prediction using machine learning. MACLEAPS [68] which is an SVM based tool from 2013 was not included as the links to the were not functional. SVM: Support Vector Machine, DNN: Deep Neural Network, SNP: Single-nucleotide polymorphism, LRP: layer-wise relevance propagation, CNN: convolutional neural network, LSTM: Long-short Term Memory, DL: Deep-learning, VCF: Variant Call Format, NN: Neural Network. RRB: ridge regression best linear unbiased prediction, BTS: bootstrap trees, RF: Random Forest, SNV: Single-nucleotide variant. All software are publicly available; COMBI: part of the GWASpi toolbox 2.0 (https://bitbucket.org/gwas_combi/gwaspi/) (Accessed on the 23 September 2023), DeepCombi: https://github.com/AlexandreRozier/DeepCombi (Accessed on the 24 September 2023), Deep Mixed Model: https://github.com/HaohanWang/DMM (Accessed on the 25 September 2023), DeepWAS: https://github.com/ cellmapslab/DeepWAS (Accessed on the 26 September 2023), GenNet: https://github.com/ArnovanHilten/GenNet (Accessed on the 28 September 2023), GMSTool: https://github.com/JaeYoonKim72/GMStool (Accessed on the 2 October 2023), GWAAN: https://github.com/hubner-lab/GWANN (Accessed on the 3 October 2023), IMEGES: https://github.com/WGLab/iMEGES (Accessed on the 4 September 2023).
